# DTW4Omics: Comparing Patterns in Biological Time Series

**DOI:** 10.1371/journal.pone.0071823

**Published:** 2013-08-20

**Authors:** Rachel Cavill, Jos Kleinjans, Jacob-Jan Briedé

**Affiliations:** Department of Toxicogenomics, Maastricht University, Maastricht, The Netherlands; Semmelweis University, Hungary

## Abstract

When studying time courses of biological measurements and comparing these to other measurements eg. gene expression and phenotypic endpoints, the analysis is complicated by the fact that although the associated elements may show the same patterns of behaviour, the changes do not occur simultaneously. In these cases standard correlation-based measures of similarity will fail to find significant associations. Dynamic time warping (DTW) is a technique which can be used in these situations to find the optimal match between two time courses, which may then be assessed for its significance. We implement DTW4Omics, a tool for performing DTW in R. This tool extends existing R scripts for DTW making them applicable for “omics” datasets where thousands entities may need to be compared with a range of markers and endpoints. It includes facilities to estimate the significance of the matches between the supplied data, and provides a set of plots to enable the user to easily visualise the output. We illustrate the utility of this approach using a dataset linking the exposure of the colon carcinoma Caco-2 cell line to oxidative stress by hydrogen peroxide (H_2_O_2_) and menadione across 9 timepoints and show that on average 85% of the genes found are not obtained from a standard correlation analysis between the genes and the measured phenotypic endpoints. We then show that when we analyse the genes identified by DTW4Omics as significantly associated with a marker for oxidative DNA damage (8-oxodG), through over-representation, an Oxidative Stress pathway is identified as the most over-represented pathway demonstrating that the genes found by DTW4Omics are biologically relevant. In contrast, when the positively correlated genes were similarly analysed, no pathways were found. The tool is implemented as an R Package and is available, along with a user guide from http://web.tgx.unimaas.nl/svn/public/dtw/.

## Introduction

Time courses provide insight into patterns and sequential biological events, and therefore temporal studies are an important tool in biological research. The systems we study are not static, but change dynamically over time. A large amount of 'omics research is currently performed by taking samples at a single time point and seeking the significantly changed genes, proteins and/or metabolites. However, given the dynamic features of biological systems we know the chosen time point will strongly influence the obtainable results. Therefore, studying the changes over time is crucial to obtain a fuller understanding of the system as a whole.

However, when studying stressor-induced biological time courses in pharmacology or toxicology one can face difficulties with the interpretation of the results due to differences in the kinetics of time courses between biological measurables. Even when aspects of the system are related and therefore display similar patterns of change over time, we expect to see delays and differences in the speed of this change. Under these circumstances standard correlation analysis can often fail to find any association between the elements, and here DTW may be used.

**Figure 1:**
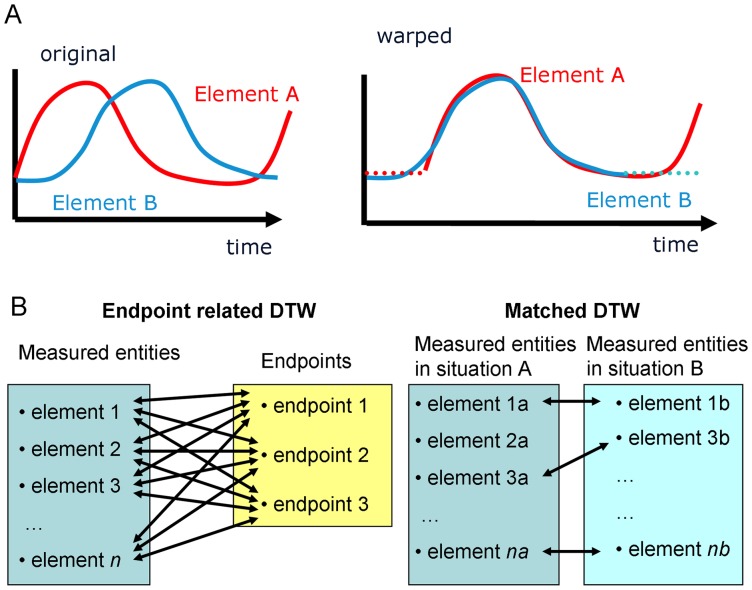
Dynamic time warping. Panel A shows how time series can be aligned using dynamic time warping from the original (left) to the warped (right). Panel B shows the two varieties of DTW provided by DTW4Omics, showing the comparistons which are considered in case.

DTW works by finding the optimal alignment between time courses [1]. As shown in figure 1A, there are often measured biological elements which follow similar patterns, but which start at different times. By introducing delays into the time courses for one or both elements (shown by dotted lines in the second panel), DTW finds an optimal match, with a bias towards matches which introduce fewer delays. In this case the delay was at the start of the time course, however delays are also allowed mid-time course and may also occur in both patterns being matched, for instance a delay may be used towards the start in one time course and another delay towards the end in the other time course. It should be noted that DTW alone gives no information about the probability of finding the matches, only on the distance between the matched time courses and the delays introduced to obtain such a match.

One common application of dynamic time warping is in speech recognition where it can automatically allow adjustment of the signal to cope with different speeds of speaking.

In the biological domain DTW has been previously used for analysing gene expression data in several studies [2]–[7]. For instance, in [3] they used it as an alternative to clustering methodologies to look for clusters of genes in a drosphilia life cycle transcriptomics dataset. They searched for clusters which showed the same pattern of expression on different timescales and were able to find clusters which were associated with different stages of the life cycle. Conversely, Hermens and Tsiporkova [7], used DTW to help them combine data from different periodic cell regulations studies in plants, where current methods to synchonise do not cover the whole cell and synchronisation deterioates rapidly meaning that later timepoints are compromised. They used DTW to align the overlapping parts of multiple experiments and thus to produce much longer datasets than it is possible to produce in a single experiment. They demonstrate that this method improves their estimates of gene-periodicity using data from arabidopsis and yeast.

Currently there is one tool for DTW, aimed specifically at analysing 'omics data, in this case global gene expression. This tool, GenTχWarper [8] provides a user interface to align sets of genes giving visualisations of the original and the warped data. However, GenTχWarper has no facility for estimating the significance of a particular alignment and therefore in their studies, comparisons between their tool and standard correlation measures were performed by comparing the biological relevance of the top 50 genes. However, in 'omics data analysis we should not be interested in the top hits from a method, but in those hits which have a significant probability of not occurring by chance. In order to allow this evaluation we developed DTW4Omics, an R-package which gives an estimate of alignment significance along with further visualisations to obtain an overview of the significantly associated entities and their warping.

Since our DTW4omics package can be generally applied to time related omics data from any source, throughout this paper and the DTW4Omics package we use the general terms ‘Endpoints’ and ‘Entities’ to refer to the different time courses which are undergoing DTW. How DTW4Omics uses endpoints and entities is shown in figure 1B and explained further in the materials and methods section. However, it is important to note that both endpoints and entities could be anything which has been measured repeatedly over a time course. For instance, if you were interested in which of a large set of metabolites had a similar time course to any of a small set of preselected genes, then the program would run best if the genes were entered as the endpoints and the metabolites as the entities. Conversely, in the case presented in this paper we use cell cycle measurements among other things as endpoints and look for sets of genes (entities) which follow these patterns.

## Materials and Methods

DTW4Omics is implemented as an R package, and utilises the DTW package [9] to perform the warping. It contains two key functions, which dictate the pairings which are examined for associations as shown in figure 1B.

The function for endpoint related DTW takes 6 parameters, 4 of which are essential. A matrix of the entities measured (n*t, where n  =  number of entities, t  =  number of time points), a matrix of the endpoints (t*e, where e  =  number of endpoints), a list of directory names where the results will be stored, a list of the numerical values for the time points and then optionally the type of scaling to apply to the data before DTW and a maximum q-value allowed for an entity to be significant. The row names of the entity matrix are used as labels in the results files and plots.

For each endpoint, the optimal matches for each entity are calculated with DTW. The order of the time points are then permuted (separately for entity and endpoint), to allow an estimation of the p-value. The p-values obtained for each entity are stored and Benjamini-Hochberg false discovery rate correction is applied to produce a list of significant entities.

**Figure 2:**
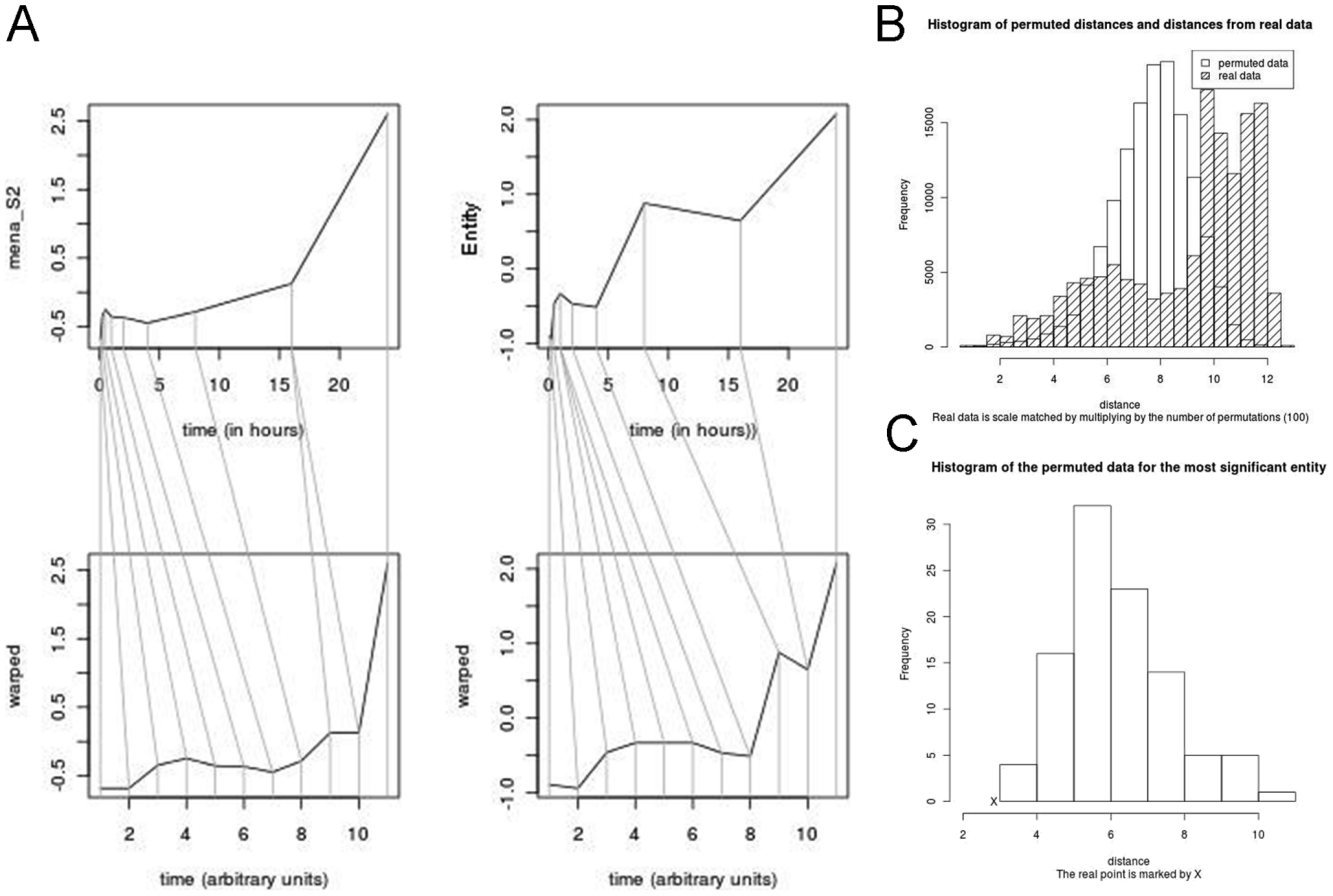
Output plots. A – showing gene and endpoint before and after warping. B – Histogram of real and permuted distances obtained through DTW. C – Histogram of the permuted distances obtained for the most significant gene, with the real distance marked by an X.

A set of jpeg image files is created storing plots of the original and warped time courses for every significant entity (see Figure 2A). Two other histograms are also produced. The first overlays two histograms, one of the optimal warped-distances between entity and endpoint generated from the real data, and the second of the distances generated from the permuted data (Figure 2B). The second histogram is similar, except it shows only the permuted distances for the most significant entity, overlaid with an X to mark the optimal distance found in the real data (Figure 2C).

Finally, there are two text files produced as output, one containing a list of the significant entities found, and the other containing a summary of the run, with details of the most significant entity found (regardless of whether it reached the specified q-value threshold).

A second function performs matched DTW which allows pairs of profiles to be compared (see figure 1B). This function is envisaged for situations where the same entities have been measured under different stressors which are expected to produce the same response but where it may occur at different delays, or where the same stressor is under investigation in different systems. In these cases, the package provides this function which compares, for instance, gene A under condition 1, with gene A under condition 2 and generates an optimal match, it then proceeds with genes B, C etc.

Taking two matrices of measurements of dimensions, n_1_*t_1_ and n_2_*t_2_, where t_1_ and t_2_ are the numbers of time points from two experiments and n_1_ and n_2_ are the number of measured entities with a significant time course. It is not necessary that the same timepoints are measured in each system, or even that the same number of time points is measured, and similarly it may be that a different overall set of entities has been measured, however the analysis will only be performed on the overlap as determined by the row labels. This is shown in figure 1B, where the list of measured entities for situation A contains element 2a, but there is no corresponding element 2b, so no DTW comparison is performed. For each entity the probability of obtaining such a match is estimated as before using perturbations of the order of the data points. Once all optimal matches are generated and the probabilities thereof are estimated a false discovery rate criterion is applied and the significant genes are reported with figures plotted per gene as per Figure 1A.

## Results and Discussion

We used data from [10] (GSE15327) on the effects of oxidative stress caused by H_2_O_2_ or menadione exposure on gene expression in human colon carcinoma cell line Caco-2. This set was generated to investigate reactive oxygen species-induced oxidative stress in the colon, which is involved in inflammatory bowel diseases and suggested to be associated with colorectal cancer risk. This set includes changes in cell cycle parameters and measurements of 8-oxo-dG (8-oxo-2-deoxyguanosine), a marker for oxidative DNA damage at each of 9 time points spread over 24 hours. First we filtered the genes to select those with no missing values and showing a significant time course, using a threshold of at least one time point having an average expression value of at least 2 standard deviations from the mean. This resulted in 1391 genes from the menadione treatment and 1292 genes from hydrogen peroxide exposure. We used the DTW4Omics tool, with unit variance scaling to rescale the genes and endpoints so that values would be comparable.

We compared the lists of significant genes generated by DTW4Omics with those genes with a significant Pearson correlation to the endpoints (Table 1). This table shows that on average 85% of genes found by DTW4Omics are not found by the standard correlation analysis and conversely it does find an average of 46% of those genes found by correlation analysis. This suggests that whilst correlation analysis is more sensitive to those genes where the time courses run in parallel, DTW4Omics can give a very significant amount of extra genes whose time courses are significantly associated with an endpoint, but which do not correlate strongly due to delays or differences in speed.

**Table 1:** Comparing DTW4Omics selected genes with correlation analysis.

Oxidant		G1	S	8-oxo-dG	Time
**Mena-dione**	No. of genes with positive correlation	34	53	51	127
	No. of genes with DTW	99	102	141	145
	Overlap with correlated genes	13	18	22	86
	Percentage of DTW genes not found by correlation	87%	82%	84%	41%
	Percentage of positive correlation genes found by DTW	38%	34%	43%	68%
**H_2_O_2_**	No. of genes with DTW	14	0	17	13
	Percentage of DTW genes not found by correlation	100%	–	100%	100%

G2 is not shown as it gave no correlated genes under any conditions and with H_2_O_2_ no genes were found with correlation so these rows are omitted.

In order to validate the biological relevance of the genes found by DTW we took the genes significantly associated by DTW between menadione exposure and 8-oxo-dG and input them into ConsensusPathDB [11] for over-representation analysis. We selected this endpoint, as we know that 8-oxo-dG is a biomarker for oxidative stress induced DNA damage, and therefore we expect that if the DTW4Omics package is working the selected genes for this endpoint will be associated with these pathways. The background list used for the over-representation analysis contained all the genes with a significant time-course and which therefore were assessed by DTW for association with 8-oxo-dG. The most significant pathway from this analysis was the Oxidative Stress pathway from wikipathways (p = 0.000555). In contrast when the positively correlated genes were input into ConsensusPathDB, no significantly associated pathways were found.

This example clearly demonstrates the ability of DTW4Omics to highlight relevant genes through the alignment of time series data, in this case between genes and phenotypical markers, thus achieving a phenotypic anchoring of 'omics responses to oxidative stress.

To test the matched entities functions we generated a set of simulated data. First we generated random time series for 11,000 entities, these time series were designed to be smooth, as opposed to a series of random values. The first 10,000 entities in the second set were generated by adding noise and time shifts to the first set. 10 levels of time shifting were used ranging from 0–9 units of shift and 10 levels of noise. The final 1,000 entities in the second set were generated at random in the same manner as those in the first set. For a full description of the generation of these data, including matlab code see Data S1.

**Figure 3:**
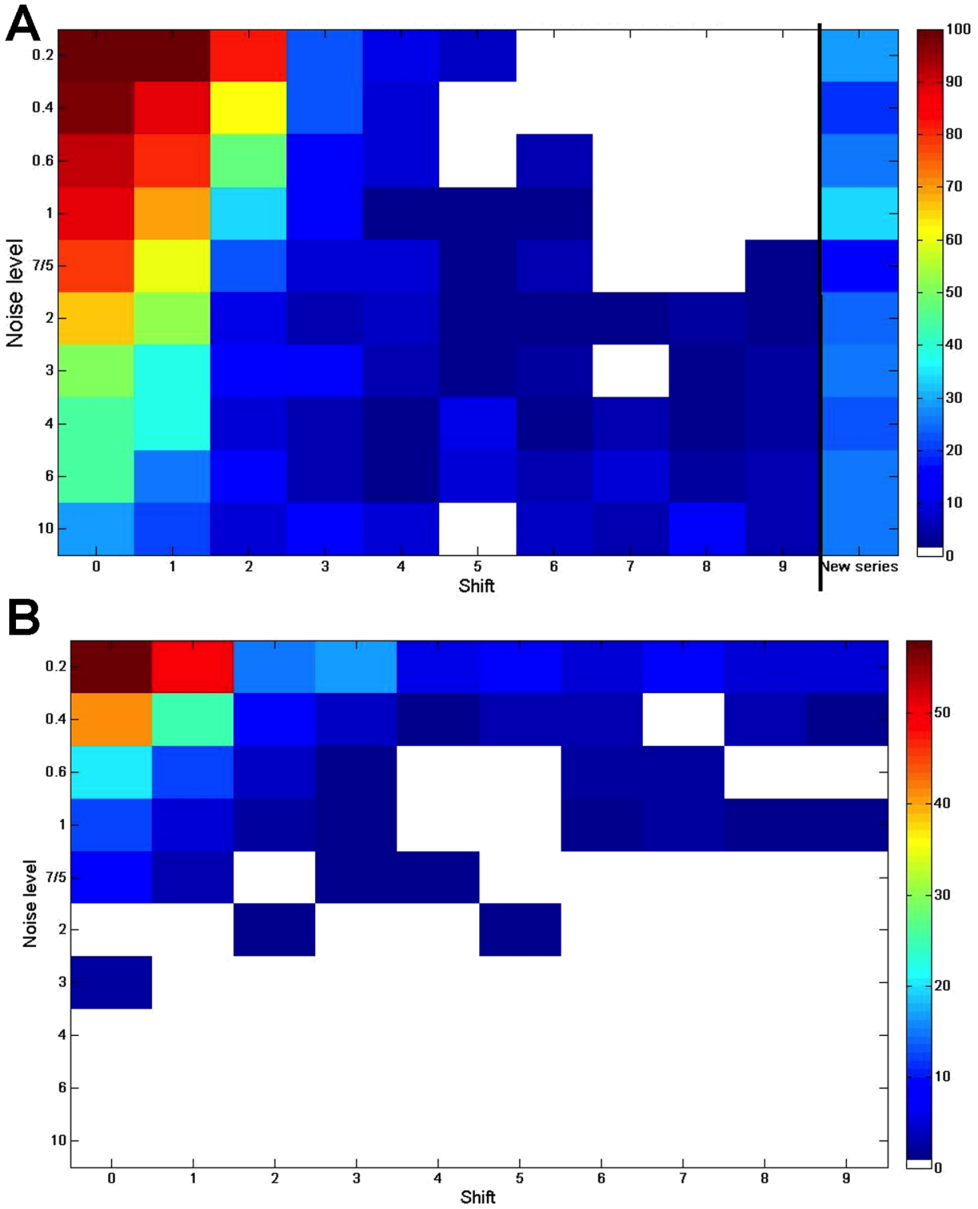
Results from the simulated data for matched DTW. Both panels show the results of the different levels of noise (low noise at top, high noise at bottom) and different amounts of time shift between the sequences (0 shift on left, 9 units shift on right). All time series were 20 units long. Panel A shows the percentage of elements which were (correctly) recognised as matches using an FDR of 5% and the matched DTW function. Panel B shows the percentage of matches where the matched pattern exactly matched the intended differences in the time courses.

The results of these simulations were measured in two ways, firstly in how many cases would the entities appear as significantly associated, given a standard FDR of 5% and secondly, how often would the correct pattern of time shifts be detected. These results, shown in figure 3 clearly show the expected pattern, that as more noise is added and the time shift increased it becomes less likely that the DTW algorithm will recognise the pair of entities as a match and discover the correct pattern of time shifts. However, this also shows that with no time shift the match will often still be made even in the presence of high levels of noise, and with small time shifts of 1 or 2 units the detection power decreases, but is still good. This may also be important to consider when designing experiments for analysis by DTW, since a set of time points sampled too closely together in time may inhibit the ability of the algorithm to find matching pairs, since the expected shift between two elements may extend to be greater than 2 time points.

The high level of similarity detection when comparing two random series can be explained by the fact that the time series were generated to be smooth, and therefore are more likely to be matched to each other than when the time points are randomly permuted in the significance estimation process. This could indicate that when longer and smoother time series from real data are analysed through this tool, the p-values may be over-estimating the significance of the matches, although it will still serve to rank the entities. Therefore, we suggest that with longer, smoother time series a stricter FDR of, for instance 1% may be applied to take into account these false positive results.

Finally, it is interesting to compare the two plots in figure 3A and 3B. 3A shows a much higher detection of similarity when there is a low amount of time shift, whereas 3B shows that the correct pattern of shift can be best detected when there are low levels of noise. This would suggest that in the presence of a reasonable level of noise entities are often correctly associated, but the wrong pattern of time shifts is assigned. Similarly in a low noise environment, the right time pattern may be determined, but the match may not be statistically significant. Since in biology we are generally working in high noise environments, either through biological reproducibility or limitations of technical reproducibility, we can conclude that the lists of selected entities are likely to be more robust than the time shift patterns generated.

We also used the matched function on the oxidative stress dataset to find if there is a set of genes which exhibited the same response pattern (perhaps at different delays) after exposure to each compound. Here we might expect to find genes linked to a common mechanism of toxicity. We found that no genes had significantly associated time courses between their expression after H_2_O_2_ and after menadione treatments. This matches the conclusions of the original paper [10] where it was found that the different oxidants induced oxidative stress through different mechanisms.

## Conclusions

We introduced a new tool for analysing time-course data, DTW4Omics which uses DTW to generate a list of genes whose time course may be minimally adjusted to obtain an optimal match to that of an endpoint. Many of the genes found in our test were not significant using correlation analysis, and thus this tool is complementary to such an analysis and we recommend applying both approaches in combination. Further the gene list generated through DTW was then able to be associated with biologically relevant pathways, in contrast to the list generated from positively correlated genes alone.

Through the presentation of simulated data we have seen that DTW can also be applied to recognise time courses which are matched between two elements in different datasets, and through exploring the consequences of adding noise and time shifts to this simulated data we have found that in high noise environments (such as those found when measuring biological systems) the detection of paired elements is likely to be more robust than the detection of the “correct” pattern of time shifts.

Whilst we have presented transcriptomics data combined with phenotypic endpoints from non-omics technologies, this approach can clearly be applied much more widely as a method for integrating data from different sources.

## Supporting Information

Data S1 **Description of the generation of simultated time-series data including matlab code.**
(DOC)
