# Supplementary material for: DTW4Omics: Comparing Patterns in Biological Time Series
Source: PLoS One. 2013 Aug 20;8(8):e71823. doi: 10.1371/journal.pone.0071823 (PMC3748037; doi:10.1371/journal.pone.0071823)
Supplement: Data S1 — Description of the generation of simultated time-series data including matlab code. (DOC) [file pone.0071823.s001.doc]

**Description of generation of simulated data:**

The simulated data for the matched DTW was produced as follows.

The dimensions of the data were 2 blocks each of 11000 elements and 20 timepoints. (11000 rows, 20 columns).

The first block and the last 1000 elements of the second block were generated completely at random according to the procedure detailed under generating a random time series.

The remaining first 10000 elements of the second block were generated based on the profiles of their matching elements from the first block, with this pattern

| **Element numbers** | **Noise added** | **Time shift** |
| --- | --- | --- |
| 1-100 | Lowest | 0 |
| 101-200 | Low | 0 |
| … | … | … |
| 901-1000 | Highest | 0 |
| 1001-1100 | Lowest | 1 |
| 1101-1201 | Low | 1 |
| … | … | … |
| 2001-2100 | Low | 2 |
| … | … | … |
| 9901-10000 | Highest | 9 |

In this way there were 100 elements with each level of noise and time shift in the set.

*Generating a random time series.*

To generate the random time series we;

- Generated random starting values uniformly distributed in the range [-10,90].
- Generated random starting slopes normally distributed mean=0, SD=1.
- Generated a binary array, to tell us when to alter the existing slope and when to use a completely new slope.
- Generated random slope values uniformly distribued [-1,1].
- Generated random second derivative values normally distributed, mean=0, SD=1.
- The first timepoint was set to be the random starting value
- The second timepoints was set to be the random starting value + random starting slope.
- For all other timepoints;
  - If the binary matrix = 1; then adjust the slope by the second derivative value and calculate new point.
  - If the binary matrix = 0; then adjust the slope by the second derivative value, multiply by the absolute value of the random slope values and then by -1.

*This gives a slight change in slope magnitude, and a change in the direction of the slope, leading to peaks and troughs reminiscent of biological data.*

*Adding noise to the first dataset*

To generate the second dataset we added increasing levels of noise to the data from the first dataset. Noise factors= [0.2, 0.4, 0.6, 1, 7/5, 2, 3, 4, 6, 10].

For each value we added a random number normally distributed, mean=0, SD=1 * noise factor to the slope for the equivalent point in datasetA, and then calculated the new point based on this new slope. This meant the differences were cumulative over the timecourse.

After this the time shifts were performed.

*Appendix – Matlab code for generating random data.*

starting_values=rand(11000,1)*100-10;

starting_slopes=randn(11000,1);

second_derivatives=randn(11000,19);

binary=round(rand(11000,19));

slopes=rand(11000,19)*2-1;

datasetA(:,1)=starting_values;

datasetA(:,2)=datasetA(:,1)+starting_slopes;

new_slope=starting_slopes;

for i=3:20

new_slope=new_slope+second_derivatives(:,i-1);

datasetA(binary(:,i-1)==1,i)=datasetA(binary(:,i-1)==1,i-1)+new_slope(binary(:,i-1)==1);

datasetA(binary(:,i-1)==0,i)=datasetA(binary(:,i-1)==0,i-1)+new_slope(binary(:,i-1)==0).*abs(slopes(binary(:,i-1)==0,i-1))*-1;

end

plot(datasetA')

for i=1:19

diffsA(:,i)=datasetA(:,i+1)-datasetA(:,i);

end

noise=[1,2,3,5,7,10,15,20,30,50];

noise=noise/5;

starting_valuesB=zeros(11000,1);

for i=1:10

for j=1:10

diffsB((i-1)*100+1+(j-1)*1000:i*100+(j-1)*1000,:)=diffsA((i-1)*100+1+(j-1)*1000:i*100+(j-1)*1000,:)+randn(100,19)*noise(i);

starting_valuesB((i-1)*100+1+(j-1)*1000:i*100+(j-1)*1000)=starting_values((i-1)*100+1+(j-1)*1000:i*100+(j-1)*1000,:)+randn(100,1)*noise(i);

end

end

datasetB=zeros(11000,19);

datasetB(:,1)=starting_valuesB;

for i=1:19

datasetB(1:10000,i+1)=datasetB(1:10000,i)+diffsB(:,i);

end

for j=1:10

datasetB((j-1)*1000+1:j*1000,:)=circshift(datasetB((j-1)*1000+1:j*1000,:),[0,j-1]);

end

starting_valuesB2=rand(1000,1)*100-10;

starting_slopesB2=randn(1000,1);

second_derivativesB2=randn(1000,19);

binaryB2=round(rand(1000,19));

slopesB2=rand(1000,19)*2-1;

datasetB2(:,1)=starting_valuesB2;

datasetB2(:,2)=datasetB2(:,1)+starting_slopesB2;

new_slopeB2=starting_slopesB2;

for i=3:20

new_slopeB2=new_slopeB2+second_derivativesB2(:,i-1);

datasetB2(binaryB2(:,i-1)==1,i)=datasetB2(binaryB2(:,i-1)==1,i-1)+new_slopeB2(binaryB2(:,i-1)==1);

datasetB2(binaryB2(:,i-1)==0,i)=datasetB2(binaryB2(:,i-1)==0,i-1)+new_slopeB2(binaryB2(:,i-1)==0).*abs(slopesB2(binaryB2(:,i-1)==0,i-1))*-1;

end

datasetB(10001:11000,:)=datasetB2;
